# Production of 3-Hydroxypropanoic Acid From Glycerol by Metabolically Engineered Bacteria

**DOI:** 10.3389/fbioe.2019.00124

**Published:** 2019-05-24

**Authors:** Carsten Jers, Aida Kalantari, Abhroop Garg, Ivan Mijakovic

**Affiliations:** ^1^Novo Nordisk Foundation Center for Biosustainability, Technical University of Denmark, Lyngby, Denmark; ^2^Department of Biomedical Engineering, Duke University, Durham, NC, United States; ^3^Systems and Synthetic Biology Division, Department of Biology and Biological Engineering, Chalmers University of Technology, Gothenburg, Sweden

**Keywords:** 3-hydroxypropanoic acid, glycerol, biosynthesis, cell factory, synthetic biology, metabolic engineering

## Abstract

3-hydroxypropanoic acid (3-HP) is a valuable platform chemical with a high demand in the global market. 3-HP can be produced from various renewable resources. It is used as a precursor in industrial production of a number of chemicals, such as acrylic acid and its many derivatives. In its polymerized form, 3-HP can be used in bioplastic production. Several microbes naturally possess the biosynthetic pathways for production of 3-HP, and a number of these pathways have been introduced in some widely used cell factories, such as *Escherichia coli* and *Saccharomyces cerevisiae*. Latest advances in the field of metabolic engineering and synthetic biology have led to more efficient methods for bio-production of 3-HP. These include new approaches for introducing heterologous pathways, precise control of gene expression, rational enzyme engineering, redirecting the carbon flux based on *in silico* predictions using genome scale metabolic models, as well as optimizing fermentation conditions. Despite the fact that the production of 3-HP has been extensively explored in established industrially relevant cell factories, the current production processes have not yet reached the levels required for industrial exploitation. In this review, we explore the state of the art in 3-HP bio-production, comparing the yields and titers achieved in different microbial cell factories and we discuss possible methodologies that could make the final step toward industrially relevant cell factories.

## Introduction

The development of microbial cell factories is fueled by aspirations to develop sustainable processes based on renewable resources. The goal is mitigation of the negative environmental consequences of production of fuels, chemicals and other materials.

**Figure 1:**
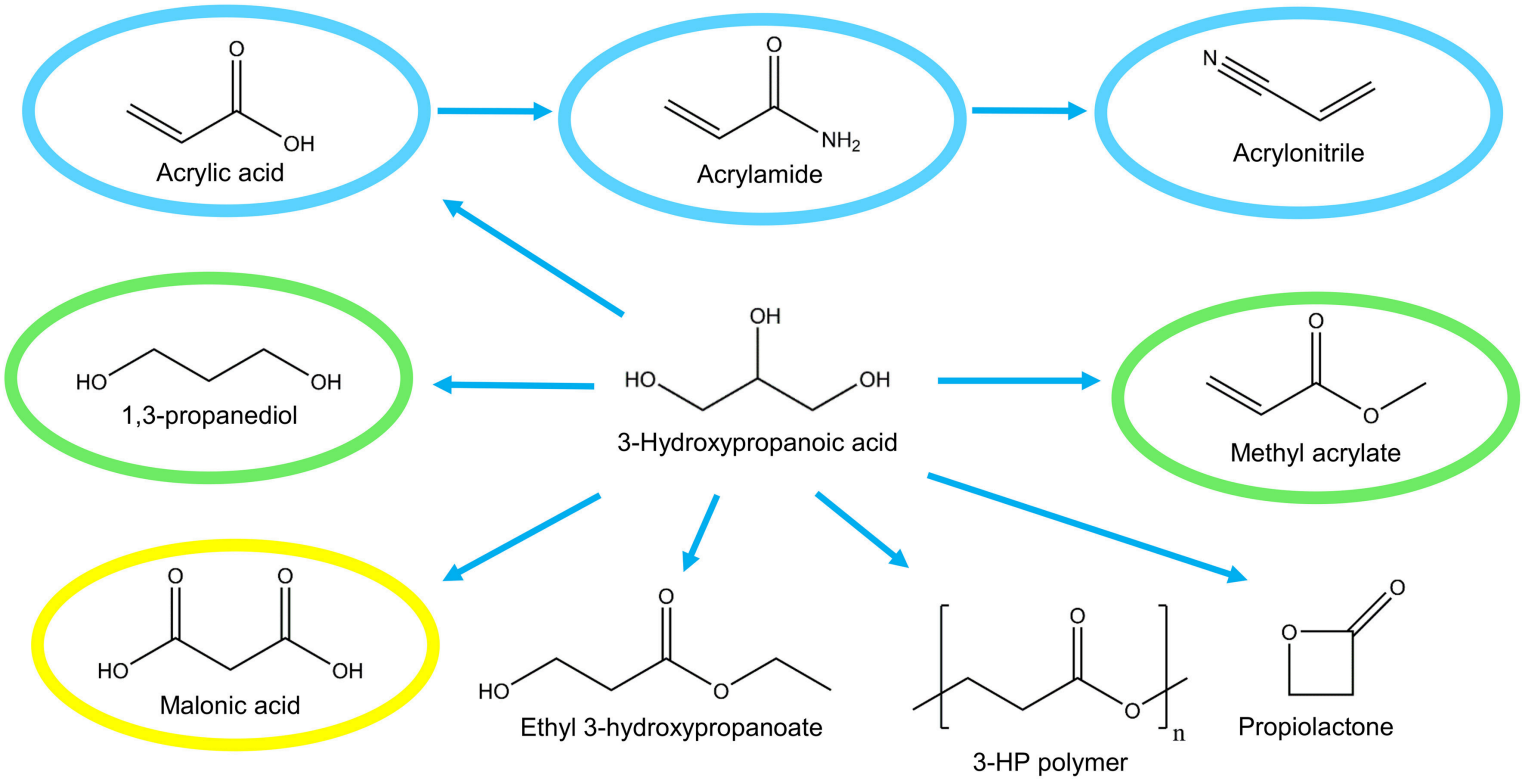
Potential industrial uses of 3-hydroxypropanoic acid. Global market of derived compounds are indicated; >1,000 M$ (blue), 100–1,000 M$ (green), <100 M$ (yellow).

The development of microbial cell factories for production of the platform chemical 3-hydroxypropanoic acid (3-HP) has attracted much attention in the last decade. 3-HP is a non-chiral (optically inactive), small three-carbon molecule and a structural isomer of lactic acid. Specifically, 3-HP is a precursor for the production of a number of valuable chemicals including acrylic acid and bioplastics (Figure 1). Bio-based production of 3-HP also has the potential to “turn waste into a resource” since several metabolic pathways exist for converting glycerol, a by-product of biodiesel production, into 3-HP.

Here, we will briefly introduce the metabolic pathways that can be used to produce 3-HP from glycerol. Based on work accumulated over the last decade, we will present current knowledge pertaining to different production hosts, enzymes, and strategies for optimization of production pathway and host metabolism as well as process engineering for attaining high-level production of 3-HP. Finally, we will discuss how recent developments in synthetic biology and metabolic engineering might form the basis for further improvements of production strains and the eventual goal of industrially viable and sustainable 3-HP production.

## Metabolic Pathways for Synthesis of 3-HP Starting From Glycerol

### Metabolic Pathways Found in Nature

**Figure 2:**
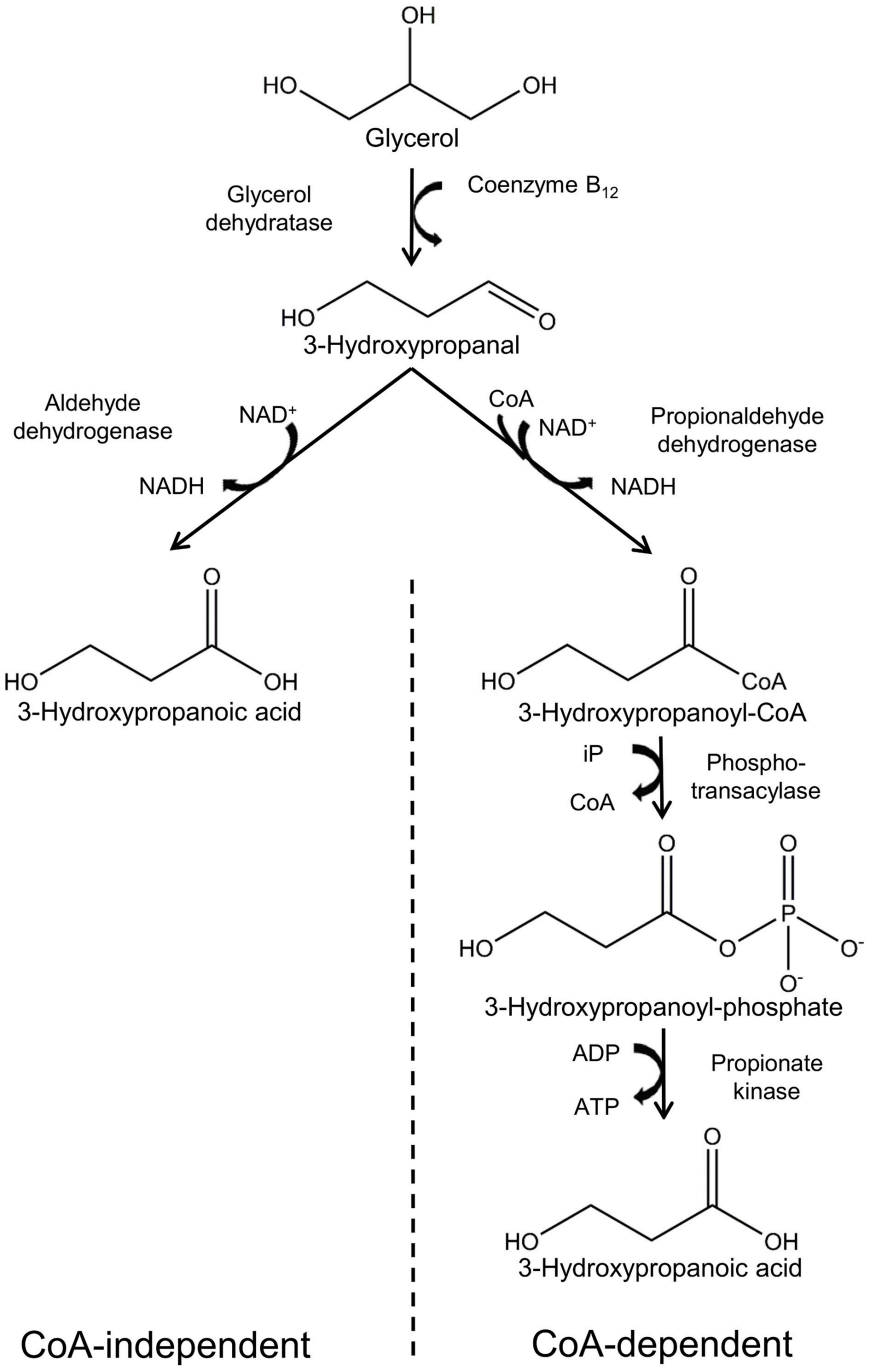
Synthesis of 3-hydropropanoic acid from glycerol via the CoA-independent and -dependent pathways.

A number of microorganisms have been reported to naturally produce 3-HP using various pathways and diverse substrates such as glycerol, glucose, CO_2_, and uracil. Several reviews have described these in detail (Kumar et al., 2013a; de Fouchécour et al., 2018), and in this review we will focus only on the pathways for which glycerol is the substrate. Two pathways are known for conversion of glycerol into 3-HP: the CoA-dependent pathway and the CoA-independent pathway (Figure 2).

The CoA-dependent pathway has been most extensively studied in *Lactobacillus reuteri* and proceeds with conversion of glycerol to 3-hydroxypropanal (3-HPA) catalyzed by coenzyme B_12_-dependent glycerol hydratase (PduCDE). 3-HPA is subsequently converted to 3-HP via 3-hydroxypropanoyl-CoA and 3-hydroxypropanoyl-phosphate, catalyzed by the enzymes propionaldehyde dehydrogenase (PduP), phosphotransacylase (PduL), and propionate kinase (PduW), respectively (Dishisha et al., 2014).

In the CoA-independent pathway, glycerol is converted to 3-HP in 2 steps. As in the case of the CoA-dependent pathway, glycerol is first converted to 3-HPA, while in the second step, 3-HPA is converted directly to 3-HP in a reaction catalyzed by aldehyde dehydrogenase (Kumar et al., 2013a). While the CoA-independent pathway is by far the most employed for production of 3-HP using engineered bacteria, it appears to have little relevance in nature. This is possibly due to low activity of aldehyde dehydrogenase in wild type bacteria (Zhu et al., 2009).

## Production Hosts

A number of microorganisms have been used for the production of 3-HP, both natural isolates as well as engineered microorganisms (Table 1). For the selection of an appropriate production host, there are several parameters to consider. The microorganism should demonstrate tolerance to organic acids, specifically 3-HP, as well as potentially toxic impurities in crude glycerol. As one of the widely used glycerol dehydratases is coenzyme B_12_-dependent, the production host should preferentially be capable of synthesizing coenzyme B_12_. Addition to the production medium instead of *in situ* synthesis would significantly increase the overall production cost.

**Table 1:** Overview of bacterial species applied for production of 3-HP.

**Bacterial species**	**Natural producer**	**B_**12**_ synthesis**
*Lactobacillus reuteri*	Yes	Yes
*Klebsiella pneumonia*	Yes	Yes
*Escherichia coli*	No	No
*Pseudomonas denitrificans*	No	Yes
*Shimwellia blattae*	No^a^	Yes
*Corynebacterium glutamicum*	No	No
*Bacillus subtilis*	No	No
*Synechococcus elongates*	No	No

a *Naturally produces 3-HPA*.

*Lactobacillus reuteri* is capable of naturally producing 3-HP from glycerol via the CoA-dependent pathway (Luo et al., 2011). In one study, three *L. reuteri* strains were evaluated, and all were found to produce 3-HP (Burgé et al., 2015). It also was shown that 3-HPA is toxic at a concentration of 5 g/L while 2.5 g/L 3-HP is not (as long as pH is maintained above 5). Notably, *L. reuteri* is capable of synthesizing coenzyme B_12_ (Burgé et al., 2015) and has been used for the bioconversion of biodiesel-derived glycerol into 3-HP (14 g/L) and 1,3-propanediol (1,3-PDO) (Dishisha et al., 2015). To our knowledge, there have been no attempts to engineer *L. reuteri* strains to improve production. Among lactic acid bacteria, production of 3-HP is not restricted to *L. reuteri*. Of 67 lactic acid bacteria isolates tested, 22 isolates belonging to *Lactobacillus diolivorans* and *Lactobacillus collinoides* were positive for 3-HP production from glycerol (Garai-Ibabe et al., 2008). Additionally, *L. diolivorans* is capable of using biodiesel-derived glycerol for the production of 3-HPA (Lindlbauer et al., 2017).

*Klebsiella pneumoniae* is a pathogenic bacterium that has been widely used for production of 3-HP. Along with *E. coli, K. pneumoniae* is one of the most frequently used hosts for strain development by genetic engineering for improved 3-HP production. In fact, the highest 3-HP titer reported, 83.8 g/L, was obtained in *K. pneumoniae* by the combining of optimized expression of aldehyde dehydrogenase (*K. pneumoniae* PuuC), blocking of lactic acid synthesis (*ldh1, ldh2*, and *pta* mutant), and optimization of the fermentation conditions (Li et al., 2016). An important asset of *K. pneumoniae* is its capability of producing coenzyme B_12_ (Luo et al., 2011).

*E. coli* is commonly used for metabolic engineering to produce a wide variety of compounds. It has also been widely used as a chassis for the production of 3-HP. The highest 3-HP titer reported in *E. coli* so far is 71.9 g/L, using a strain in which, besides introduction of glycerol dehydratase and aldehyde dehydrogenase, the central metabolism was modified to reduce by-product formation (Chu et al., 2015). It has been indicated that careful choice of the strain could be of importance. A comparison of nine *E. coli* strains in which a heterologous pathway for 3-HP synthesis was introduced, demonstrated differences in 3-HP production, as well as in enzyme level and activity (Sankaranarayanan et al., 2014). A drawback of using *E. coli* is the fact that it is not naturally capable of producing coenzyme B_12_. However, insertion of the *Pseudomonas denitrificans* genes for coenzyme B_12_ synthesis (more than 25 genes in 6 operons) on three plasmids led to the production of coenzyme B_12_ under both anaerobic and aerobic conditions (Ko et al., 2014).

While *K. pneumoniae* and *E. coli* are by far the most widely used production hosts, a number of other bacteria have been engineered for 3-HP production as well. *P. denitrificans* synthesizes coenzyme B_12_ in aerobic conditions where NAD(P)^+^ is efficiently regenerated (Zhou et al., 2013). Introduction of glycerol dehydratase and glycerol dehydratase reactivase from *K. pneumoniae* allowed for the production of 3.4 g/L 3-HP. By further introducing the *K. pneumoniae* aldehyde dehydrogenase, the yield increased to 4.9 g/L (Zhou et al., 2013).

Another bacterium able to synthesize coenzyme B_12_, *Shimwellia blattae*, is a 1,3-PDO producer using a native coenzyme B_12_-dependent glycerol dehydratase. Introduction of various genes including aldehyde dehydrogenase from *Pseudomonas putida* KT2442 enabled the production of 0.26 g/L Poly-3HP using crude glycerol from biodiesel production as the substrate (Heinrich et al., 2013).

*Corynebacterium glutamicum* was engineered to produce 3-HP from glucose and xylose. By a combination of efforts, an impressive titer of 62.6 g/L 3-HP was obtained in a fed-batch fermentation (Chen et al., 2017). The steps taken to achieve this titer included enabling efficient production of glycerol from glucose, introduction of the *K. pneumoniae pduCDEGH* genes encoding diol dehydratase and its reactivase and *Cupriavidor necator* aldehyde dehydrogenase and modification in sugar uptake and glycolytic flux. In *Bacillus subtilis*, introduction of glycerol dehydratase along with its reactivases, and aldehyde dehydrogenase from *K. pneumoniae*, enabled the production of 3-HP from glycerol. Upon inactivation of glycerol kinase and optimization of growth conditions, a 3-HP titer of 10 g/L was obtained in a shake flask culture (Kalantari et al., 2017). A drawback of both of these bacteria is their inability to synthesize coenzyme B_12_, which thus has to be supplemented to the production medium.

In an engineered cyanobacterium *Synechococcus elongatus* CO_2_ was converted via glycerol to 3-HP, albeit at very low titer (31.7 mg/L). *Synechococcus elongatus* is unable to synthesize coenzyme B_12_, but the problem was alleviated by producing 3-HP under anaerobic conditions using the oxygen-sensitive B_12_-independent glycerol dehydratase from *Clostridium butyricum* (Wang et al., 2015). The use of glycerol as an additional carbon source in a strain engineered to assimilate glycerol has been suggested for increasing productivity (Kanno and Atsumi, 2017).

## The Suite of Enzymes Used in Heterologous 3-HP Synthesis Pathways

As mentioned above, there are two principal pathways for production of 3-HP from glycerol. In both CoA-dependent and -independent pathways, the initial step is the conversion of glycerol to 3-HPA. In the CoA-dependent pathway, 3-HPA is converted via 3-hydroxypropanoyl-coenzyme A (3-HP-CoA) and 3-hydroxypropanoyl-phosphate (3-HP-P) to 3-HP, catalyzed by propionaldehyde dehydrogenase, phosphotransacylase, and propionate kinase, respectively (Dishisha et al., 2014). In the CoA-independent pathway, 3-HPA is converted directly to 3-HP by the action of aldehyde dehydrogenase (Kumar et al., 2013a). Both of the pathways have characteristics that are of importance for the design of cell factory and process conditions, for example, oxygen-sensitivity of enzymes and the need for co-factors such as coenzyme B_12_, and NAD^+^. In the following, we will describe the enzymes that make up these two pathways. Where available, kinetic data for the enzymes is presented in Table 2, to facilitate comparison.

**Table 2:** Summary of characterized enzymes of relevance in 3-HP production.

**Source**	**gene / protein**	***K*_**M**_ (mM)**	***V*_**max**_ (U/mg)**	***k*_**cat**_ (s^**−1**^)**	***k*_**cat**_/*K*_**M**_ x 10^**3**^ (M^**−1**^S^**−1**^)**	**Other characteristics**	**References**
**B12-dependent glycerol/diol dehydratase**							
*K. pneumoniae*	*dhaB123*	n.a.	n.a.	n.a.	n.a.		
*K. pneumoniae*	*pduCDE*	n.a.	n.a.	n.a.	n.a.		
*L. reuteri*	*pduCDE*	0.60	130.1	n.a.	n.a.	pH_opt_ 8.5	Qi et al., 2006
*C. freundii*	*gldABC*	0.59	142.9	n.a.	n.a.	pH_opt_ 8.5	Qi et al., 2006
*L. brevis*	*dhaB*	n.a.	n.a.	n.a.	n.a.	pH_opt_ 7.0	Kwak et al., 2013
**B12-independent glycerol dehydratase**							
*C. butyricum*		n.a.	n.a.	n.a.	n.a.	B_12_-independent	
**Propionaldehyde dehydrogenase**							
*L. reuteri*	*pduP*	1.18	0.35	n.a.	n.a.	broad substrate specificity	Luo et al., 2011
		n.a.	n.a.	n.a.	n.a.	Inhibition by 3-HPA (from 7 mM). NAD^+^ preference	Sabet-Azad et al., 2013
**Aldehyde dehydrogenase**							
*E. coli* K-12	*aldH*	n.a.	n.a.	28.5	58.6	NAD^+^ preference	Jo et al., 2008
		n.a.	38.16	n.a.	57.28		Chu et al., 2015
		0.49	38.10	n.a.	n.a.		Ko et al., 2012
*E. coli J*M109	*aldA*	0.31	28.4	n.a.	n.a.	NAD^+^ preference	Zhu et al., 2009
*C. necator*	*gabD4*	n.a.	55.12	n.a.	71.48		Chu et al., 2015
*C. necator*	*gabD4* E209Q/E269Q	n.a.	78.07	n.a.	162.34		Chu et al., 2015
*K. pneumoniae DSM 2026*	*puuC*	0.48	22.25	19.81	41.44	pH_opt_ 8.0	Raj et al., 2010
		0.48	27.73	n.a.	n.a.		Ko et al., 2012
**A. brasilense**	KGSADH	1.6	n.a.	15	9)	NAD^+^ preference	Park et al., 2017
**A. brasilense**	KGSADH 108-QR	0.17	n.a.	6	35	NAD^+^ preference	Park et al., 2017
**Aldehyde oxidase**							
*Pseudomonas sp. AIU 362*	*aloD*	6.7	41.8	n.a.	n.a.	NAD^+^-independent	Li et al., 2014

### Glycerol and Diol Dehydratase

#### Coenzyme B_12_-Dependent Dehydratases

Coenzyme B_12_-dependent glycerol dehydratase (EC 4.2.1.30) and diol dehydratase (EC 4.2.1.28) are isofunctional enzymes that catalyse the dehydration of 1,2-diols to the corresponding aldehyde (e.g., glycerol to 3-HPA). Both glycerol dehydratase and diol dehydratase are composed of three subunits that form a dimer of a heterotrimer (α_2_β_2_γ_2_; Shibata et al., 1999; Yamanishi et al., 2002). In mycobacteria, the α and β subunits are fused in a single polypeptide (Liu et al., 2010). Both of the enzymes catalyze a radical process in which an adenosyl radical is formed by homolytic cleavage of the Co-C bond in coenzyme B_12_ (Daniel et al., 1998). The adenosyl radical abstracts a hydrogen from the substrate, glycerol, to generate a substrate radical. The substrate radical, upon rearrangement, re-abstracts a hydrogen to form the final product and regenerate coenzyme B_12_ (Daniel et al., 1998). If a radical side reaction takes place, coenzyme B_12_ is not regenerated, and instead, a catalytically inactive cobalamin species is formed, which binds tightly to the enzyme and thereby inactivates it (Toraya, 2003). Radical side reaction can be induced by both glycerol and oxygen, making the enzyme sensitive to oxygen (Wei et al., 2014). The inactive hydratase can be re-activated by the action of glycerol/diol dehydratase reactivase in the presence of ATP and intact coenzyme B_12_ (Mori and Toraya, 1999). Glycerol/diol dehydratase reactivase consists of two subunits that form a heterotetramer (α_2_β_2_; Liao et al., 2003). The reactivase binds ATP and hydrolyzes it to ADP. The ADP-bound reactivase forms a complex with the inactivated glycerol dehydratase. This leads to release of the damaged coenzyme B_12_. Subsequently, reactivase binds ATP and is released from the hydratase, which in turn binds coenzyme B_12_ to regenerate the active form of the enzyme (Toraya, 2003).

From the above, it can be deduced that the expression of five proteins is necessary for the efficient catalysis of glycerol to 3-HPA. In the context of engineering microorganisms for 3-HP production, the most widely used glycerol dehydratase is that of *K. pneumoniae* (*dhaB123*, and *gdrAB*), which has been used in *E. coli* (Rathnasingh et al., 2009), *K. pneumoniae* (Wang et al., 2013), *B. subtilis* (Kalantari et al., 2017), and *S. elongatus* (Wang et al., 2015). The use of other dehydratases has also been reported. In *E. coli*, the glycerol dehydratase from *Lactobacillus brevis* encoded by *dhaB123* and its reactivase *dhaR12* were used (Kwak et al., 2013). The diol dehydratase (*pduCDE*) and activator (*pduGH*) from *K. penumoniae* were used in *C. glutamicum* (Chen et al., 2017).

Considering the problem of enzyme instability, it is somewhat surprising that relatively few glycerol dehydratases have been tested. These enzymes have, by now, been discovered in many bacteria and this should provide a resource for enzyme discovery endeavors. In fact, for three selected glycerol dehydratases, the α subunit was systematically swapped, which led to identification of several combinations with improved stability and activity (Qi et al., 2006). Interestingly, fusion of the α and β subunit of *K. pneumoniae* glycerol dehydratase led to an increase in the *k*_cat_ (albeit with concomitant increase in *K*_M_; Wang et al., 2009). As mentioned, mycobacteria contain a glycerol dehydratase variant in which the α and β subunits are fused, but to our knowledge they have not been characterized or used for 3-HP production. Enzyme engineering could also prove beneficial for generation of enzyme variants with improved properties such as increased stability and activity. As an example, by rational engineering, improved resistance to mechanism-based inactivation was conferred to a glycerol dehydratase from *Klebsiella oxytoga* (Yamanishi et al., 2012).

#### Coenzyme B_12_-Independent Glycerol Dehydratase

This class of glycerol dehydratases is of particular interest since it negates the need for the rather costly coenzyme B_12_. This enzyme also performs a radical catalysis, but instead of coenzyme B_12_, it uses S-adenosylmethionine as a co-factor (Raynaud et al., 2003). This glycerol dehydratase is a homodimer and it is thus structurally simpler than its coenzyme B_12_-dependent counterpart that is composed of three subunits (Raynaud et al., 2003). While it would seem the obvious enzyme of choice, it is important to note that it is extremely sensitive to oxygen and its use requires production under strict anaerobic conditions (Raynaud et al., 2003).

Considering the fact that coenzyme B_12_ is not produced in most of the microorganisms that have been used as potential 3-HP production hosts, the use of a coenzyme B_12_-independent dehydratase would be desirable. So far, its use has only been reported in a few studies. The *C. butyricum* glycerol dehydratase was used in the cyanobacterium *S. elongatus* for production of 3-HP under anaerobic conditions. As expected, it was not functional under aerobic conditions (Wang et al., 2015). In case of *E. coli*, the coenzyme B_12_-independent dehydratase was used in the context of 1,3-PDO production. Although the reported 1,3-PDO production was relatively low, an accumulation of 3-HPA was observed, thus demonstrating functionality of the glycerol dehydratase (Dabrowski et al., 2012). Furthermore, it was used for engineering *E. coli* to produce poly (3-Hydroxypropanoate) (Andreeßen et al., 2010). The scarcity of reported use of this enzyme could be due to difficulties in engineering 3-HP producing strains using this dehydratase. Alternatively, and perhaps more likely, is that reports are scarce because of the requirement of strict anaerobic conditions for the enzyme to be functional, which is not compatible with growth of most production microorganisms.

### Enzymes for Converting 3-HPA to 3HP

#### Enzymes in the CoA-Dependent Pathway

In the CoA-dependent pathway, 3-HPA is converted via 3-HP-CoA and 3-HP-P into 3-HP. This requires three enzymes: propionaldehyde dehydrogenase, phosphotransacylase, and propionate kinase, respectively (Dishisha et al., 2014). The propionaldehyde dehydrogenase requires the cofactor NAD^+^, while one ATP is generated in the conversion of 3-HP-P to 3-HP. This pathway has been described in several organisms including *L. reuteri* and *K. pneumoniae* (Luo et al., 2012; Dishisha et al., 2014). The propionaldehyde dehydrogenase PduP from *L. reuteri* has been purified and characterized *in vitro* and shown to exhibit activity toward a broad spectrum of aldehydes, including 3-HPA, using either NAD^+^ (preferred) or NADP^+^ as a cofactor (Luo et al., 2011). *Lactobacillus reuteri* PduP was also reported to display substrate inhibition for 3-HPA (at 7 mM; Sabet-Azad et al., 2013).

The CoA-dependent pathway has been used in several engineered bacteria. The *K. pneumoniae pduPLW* genes were introduced in an *E. coli* strain harboring the *K. pneumoniae* glycerol dehydratase. Here they were shown to be functional (although the obtained titer was lower than that obtained using an aldehyde dehydrogenase from *Azospirillum brasilense*; Honjo et al., 2015). In *K. pneumoniae*, overexpression of the first gene in the pathway, *pduP*, led to a 4-fold (0.72 g/L) increase in the 3-HP titer (Luo et al., 2011). It could be speculated that the native *K. pneumoniae* CoA-dependent pathway enzymes were responsible for the conversion of 3-HP-CoA to 3-HP, and that over-expression of all three genes in the pathway would further increase the titer.

It remains to be seen whether the CoA-dependent pathway could be an alternative to the more widely used aldehyde dehydrogenases for the conversion of 3-HPA to 3-HP. Although both pathways use an NAD(P)^+^, ATP is generated in the CoA-dependent pathway. However, this should be balanced against the metabolic burden of over-expressing three enzymes instead of one. Further, it is unclear if the substrate inhibition exhibited by *L. reuteri* PduP at a relatively low concentration of 3-HPA is a general trait of propionaldehyde dehydrogenase, or whether a more robust enzyme can be discovered or engineered.

#### Aldehyde Dehydrogenase

Aldehyde dehydrogenase (EC 1.2.1.3) has decidedly been the enzyme of choice for conversion of 3-HPA to 3-HP in engineered cells. It catalyses the conversion of an aldehyde to its corresponding carboxylic acid using NAD(P)^+^ as a co-factor. In the context of the 3-HP production pathway, it is of utmost importance that enzyme activity is sufficient to ensure that no accumulation of the toxic intermediate 3-HPA takes place. Aldehyde dehydrogenases are present in most organisms, but generally their activity is not high enough to sustain a high production of 3-HP (Raj et al., 2008; Zhu et al., 2009). Consequently, many studies have focused on discovering suitable aldehyde dehydrogenases. Earlier studies mainly employed the aldehyde dehydrogenase AldH from *E. coli* K-12 in both, *E. coli* and *K. pneumoniae* (Raj et al., 2008; Zhu et al., 2009). In a study by Chu and co-workers, an indirect comparison (based on 3-HP production in *E. coli*) was made amongst 17 aldehyde dehydrogenases from various organisms, benchmarked against *E. coli* AldH. One aldehyde dehydrogenase performing better than AldH, GabD4 from *C. necator*, was identified (Chu et al., 2015). The enzyme was further improved by enzyme engineering, substantially increasing its *V*_max_ and *k*_cat_/*K*_M_, and used for high-titer production of 3-HP in both *E. coli* (71.9 g/L) and *C. glutamicum* (62.6 g/L) (Chu et al., 2015; Chen et al., 2017).

The α-ketoglutaric semialdehyde dehydrogenase (KGSADH) from *A. brasilense* has also been used in several studies. KGSADH was evaluated against *E. coli* AldH and PuuC from *K. pneumoniae* (Ko et al., 2012). This study indicated higher activity (*V*_max_) of KGSADH in extracts of the *K. pneumoniae* strain, while in contrast it had the lowest *V*_max_ and affinity for 3-HPA when enzymes purified from *E. coli* were evaluated (Ko et al., 2012). Whether this discrepancy is due to e.g., stability *in vivo* is unclear. The structure of KGSADH has been solved and has provided a basis for rational enzyme engineering. This has yielded enzyme variants with improved activity toward 3-HPA (Park et al., 2017; Son et al., 2017; Seok et al., 2018).

PuuC from *K. pneumoniae* has also been used in several production hosts, including *K. pneumoniae* and *B. subtilis* (Li et al., 2016; Kalantari et al., 2017). Substrate specificity was evaluated against 3-HPA and three other aldehydes indicating broad substrate specificity (and lowest activity when 3-HPA was used; Raj et al., 2010). Comparative studies indicated that *E. coli* AldH and *C. necator* GabD4 E209Q/E269Q were better alternatives for converting 3-HPA to 3-HP (Huang et al., 2012; Chen et al., 2017). Nevertheless, PuuC was successfully used in an engineered *K. pneumoniae* strain that produced 3-HP with a titer of 83.8 g/L (highest reported) in a bioreactor (Li et al., 2016).

Several other aldehyde dehydrogenases have been tested in the context of 3-HP production. *E. coli* YneI was shown to selectively target 3-HPA over other aldehydes (Luo et al., 2013), although it was not tested against succinic semialdehyde which was previously reported as its primary substrate (Kurihara et al., 2010). The aldehyde dehydrogenase DhaS from *B. subtilis* has also been suggested as an aldehyde dehydrogenase that is specific for 3-HPA (Su et al., 2015).

Due to the oxygen-sensitivity of glycerol dehydratase, 3-HP production is often performed under anaerobic or microaerophilic conditions, where the regeneration of NAD^+^ is diminished. Thus, efficient 3-HP production becomes a compromise between assuring optimal glycerol dehydratase activity (low oxygen level) and NAD^+^-dependent aldehyde dehydrogenase activity (high oxygen level). An interesting strategy was suggested to overcome the limitation imposed by NAD^+^, namely, the use of an aldehyde oxidase in place of dehydrogenase. Li and co-workers characterized the NAD^+^-independent aldehyde oxidase from *Pseudomonas* sp. AIU 362 and found it to exhibit a broad substrate specificity but relatively low affinity for 3-HP. When expressed in *K. pneumoniae*, a relatively low 3-HP titer was obtained (Li et al., 2014). As can be seen in Table 2, the affinity of aldehyde oxidase to 3-HPA is several-fold lower than the described aldehyde dehydrogenases, with a *K*_M_ in the range where 3-HPA becomes toxic to the cell. Considering the importance of maintaining a low concentration of 3-HPA, better enzymes would likely be needed to successfully exploit aldehyde oxidase for 3-HP production. To the best of our knowledge, no other attempts have been made at identifying a more suitable aldehyde oxidase for the conversion of 3-HPA to 3-HP.

As outlined in this section, many enzyme variants have been used in the design of 3-HP-producing strains. While some of the enzymes have been characterized biochemically, it is obvious from Table 2 that this is not a norm in the field. Nevertheless, a better understanding of the catalytic properties of these enzymes could provide a platform for more optimal selection of enzymes for constructing 3-HP pathways.

## Strain Engineering

There are several parameters to consider when engineering bacteria to produce 3-HP. As covered in sections Production Hosts and The Suite of Enzymes Used in Heterologous 3-HP Synthesis Pathways, a suitable host should be established and in most cases some or all of the enzymes needed for converting glycerol to 3-HP should be introduced. This provides a basis for 3-HP production, but is not enough to ensure the desired high titers. There are several hurdles that need to be overcome, and these include, but are not limited to, proper balancing of enzyme activities to prevent buildup of the toxic intermediate 3-HPA, efficient channeling of substrate into product, co-factor regeneration, prevention of by-product formation and countering of stress. In the following, we will explore challenges and strategies that have been applied in the engineering of strains for 3-HP production.

### Improving 3-HP Production by Optimizing Expression of the Production Pathway

As illustrated above, a number of different enzymes have been used for the construction of synthetic operons to confer the ability to produce 3-HP to various bacteria. In a number of these studies, it was shown that further optimization of the expression of the pathway genes could enhance 3-HP production.

A critical parameter is to ensure that the intermediate product 3-HPA is kept at a low concentration. 3-HPA (also known as reuterin) is a broad range antimicrobial compound. Its minimal inhibitory concentrations for various bacteria are in the range of <1.9–50 mM, and it exerts its effect via modification of thiol groups of proteins and small molecules (Cleusix et al., 2007; Schaefer et al., 2010). Considering the fact that 3-HPA is toxic to bacteria even at minute concentrations, it is evident that balancing enzyme activities to prevent 3-HPA accumulation is critical. To this end, different approaches have been attempted to fine-tune the expression levels of glycerol dehydratase and aldehyde dehydrogenase. The order in which the genes encoding aldehyde dehydrogenase and glycerol dehydratase are arranged was shown to impact 3-HP production. The rationale here is that the gene adjacent to the promoter in an operon is normally more expressed. Specifically, the arrangement favoring aldehyde dehydrogenase expression led to a higher 3-HP production due to the diminished build-up of 3-HPA (Li et al., 2013a). Different promoters for driving the expression of aldehyde dehydrogenase gene has also been successfully evaluated (Li et al., 2016).

While these approaches are simple, they do not offer a high degree of tunability, and a number of more advanced approaches have been applied for optimization of expression levels. Optimization of expression level can be done by applying various 5′ untranslated regions (5′UTRs) in the genetic constructs, which leads to differences in the translation rate. Using this approach, a better balance of the enzyme activities was obtained by fine-tuning expression of *dhaB1* encoding a glycerol dehydratase subunit. Testing only four different UTRs led to construction of a strain that enabled a 2.4-fold improvement of 3-HP titer in shake flask experiments (Lim et al., 2016). Several tools are available for the prediction of translation rate of 5′UTRs, including the UTR Designer (Seo et al., 2013), and the RBS Calculator v2.0 (Espah Borujeni et al., 2014). To the same end, a study showed potential in tuning translation by modulation of the Shine-Dalgarno sequence for which an online tool, EMOPEC, is available (Bonde et al., 2016). For a selection of 106 Shine-Dalgarno sequences, the measured protein level was within 2-fold of the predicted level in 91 % of cases (Bonde et al., 2016). While *in silico* prediction of expression level is a very attractive venue, it should be noted that even for the good predictors, the number of false predictions appear to be significant (Bonde et al., 2016). Nevertheless, these computational approaches should allow for design and evaluation of smaller, more focused libraries, compared to random mutagenesis libraries.

In most studies, researchers have taken advantage of plasmids for the expression of relevant genes in the production host. While these studies provide proof of principle, they might not be optimal for industrial production due to the well-known problem of plasmid instability in bioreactors (Gao et al., 2014). Consequently, the generation of genetically stable strains where expression cassettes are integrated into the genome will be a necessity. Additionally, the elimination of plasmids might improve the production via a reduced metabolic burden associated with plasmid maintenance and replication (Silva et al., 2012). To construct a plasmid-free strain, the *E. coli aldH* was inserted in the genome of *K. pneumoniae*. While 3-HP production was improved (over parent strain) the 3-HP titer upon production in shake flasks was low (Wang and Tian, 2017). In *B. subtilis*, a plasmid-free strain allowed the production of 3-HP in shake flasks with a titer of 10 g/L (Kalantari et al., 2017).

Considering that both multicopy vectors and strong promoters have been used for the expression of relevant genes, one foreseeable difficulty in shifting to a single (or a few) genome-integrated expression cassette will be to ensure sufficient protein synthesis in the cell. A recent study described an approach based on translational coupling between the gene of interest and an antibiotic resistance cassette, thus making a high rate of translation selectable via an increased resistance to an antibiotic (Rennig et al., 2018). With limited screening, the production of two selected proteins (a nanobody and an affibody) in *E. coli* was improved 2- and 10-fold, respectively (Rennig et al., 2018). The methodology was shown to also work in Gram-positive bacteria *B. subtilis* and *Lactococcus lactis* where production of a sialidase and a tyrosine ammonia lyase was improved 2- and 8-fold, respectively (Ferro et al., 2018).

### Engineering Host Metabolism for Improving 3-HP Production

Besides integrating an efficient pathway for the conversion of glycerol to 3-HP, it is often necessary to further modulate the host metabolism, in order to direct the substrate into the production pathway more efficiently, eliminate unwanted by-products and/or reduce stress.

**Figure 3:**
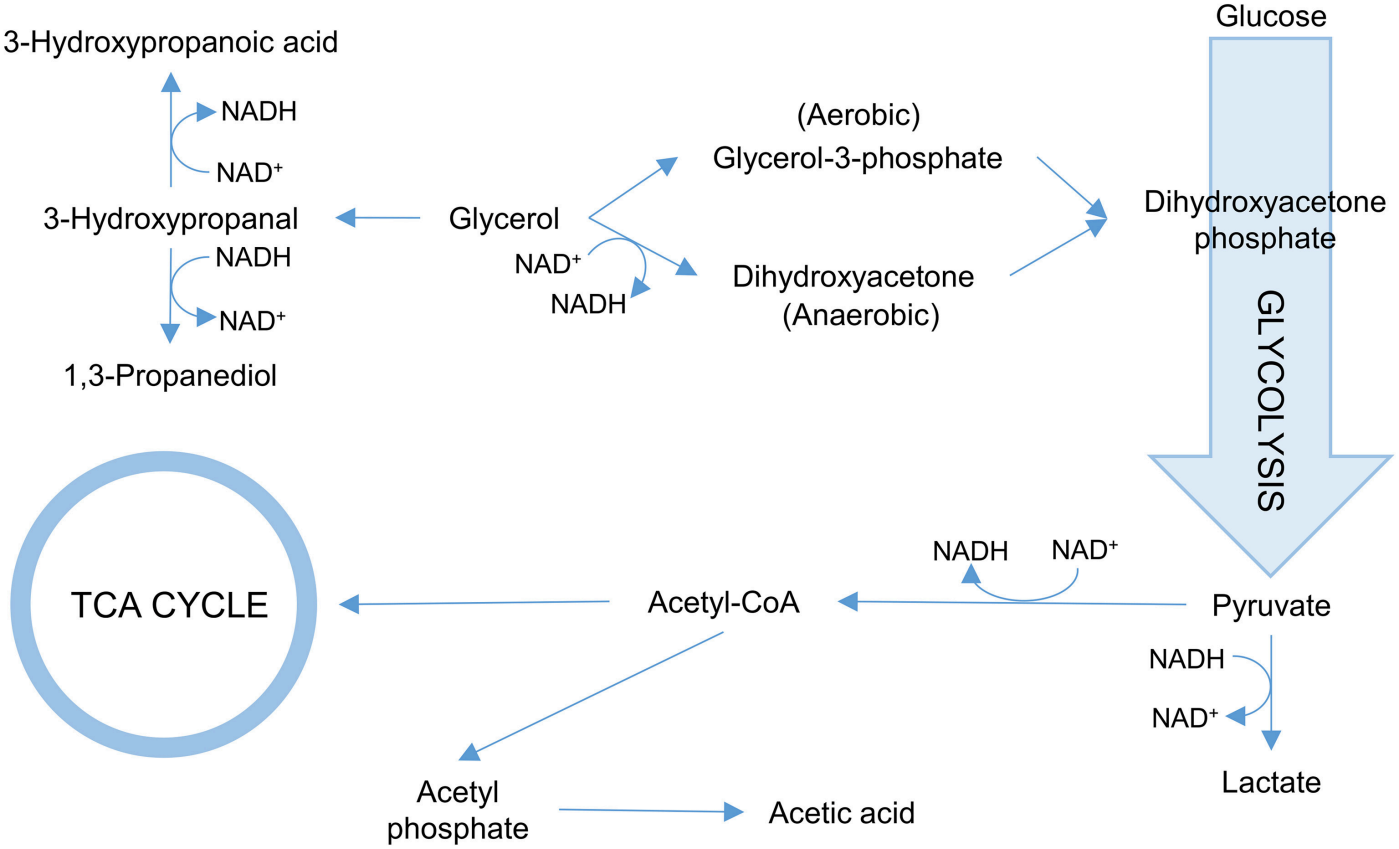
Overview of the glycerol metabolism in bacteria. Reactions consuming NADH/NAD^+^ are indicated.

One of the more common approaches is to ensure that glycerol is converted more efficiently into 3-HP. To this end, targeting the first step in the glycerol utilization pathways is common. In *E. coli*, two pathways for glycerol utilization exist. Glycerol is converted to the glycolytic metabolite dihydroxyacetone phosphate either via glycerol-3-phosphate catalyzed by glycerol kinase under aerobic conditions or via dihydroxyacetone catalyzed by glycerol dehydrogenase under anaerobic conditions (Durnin et al., 2009; Figure 3). Inactivating the gene *glpK* (which encodes glycerol kinase in *E. coli*) increased the titer of 3-HP 1.6-fold in an aerobic fed batch fermentation when co-fed with glucose and glycerol. Under the conditions used (aerobic), further inactivating the gene encoding glycerol dehydrogenase had no effect (Kim et al., 2014). Similarly, the 3-HP titer increased in *B. subtilis* co-fed on glycerol and glucose upon inactivation of *glpK* (Kalantari et al., 2017). Inactivation of *glpK* is possible in process setups where glycerol is not the sole carbon source. When cells grow only on glycerol, targeting *glpK* becomes less trivial as the fluxes toward biomass accumulation and 3-HP production need to be balanced. In *E. coli*, the *glpK* was placed under control of an inducible promoter and fine-tuning of its expression led to an increase in both 3-HP titer and yield on glycerol (Jung et al., 2014). In that study, the gene *glpF* encoding glycerol facilitator was also overexpressed to increase the influx of glycerol in the cell, which led to a modest increase in 3-HP production (Jung et al., 2014). Interestingly, the opposite approach was evaluated by Su and co-workers. They overexpressed glycerol dehydrogenase in *K. pneumoniae* to stimulate growth, and found that this strain not only exhibited faster initial growth and similar final biomass yield while using less glycerol, but also exhibited increased 3-HP production (Su et al., 2014).

In natural 3-HP producers such as *K. pneumoniae* and *L. reuteri*, 3-HPA can be oxidized to 3-HP and reduced to 1,3-PDO (Zhu et al., 2009; Dishisha et al., 2014). The conversion of 3-HPA to 1,3-PDO is catalyzed by an oxidoreductase. In *K. pneumoniae*, the most important oxidoreductase appears to be the 1,3-propanediol reductase encoded by *dhaT*. However, significant amounts of 1,3-PDO are still produced in the *dhaT* mutant, indicating that other oxidoreductases in the cell can also catalyse this reaction (Ko et al., 2012). In a subsequent attempt to eliminate 1,3-PDO formation, four oxidoreductases were inactivated, but the quadruple mutant still retained the ability to produce 1,3-PDO (Ko et al., 2015). With respect to cofactor balance, 3-HP synthesis requires NAD^+^ while 1,3-PDO formation requires NADH and thus regenerates NAD^+^. Thus, the elimination of 1,3-PDO formation adversely affects the NAD^+^/NADH balance, and in fact, it was reported that the *dhaT* mutation under 3-HP production conditions impeded cell growth (Ko et al., 2012). In *E. coli*, 3-HPA is also converted to 1,3-PDO by the action of a broad-range aldehyde oxidoreductase encoded by *yqhD*. While the deletion of *yqhD* dramatically reduces 1,3-PDO formation, it is also here not fully abolished, likely due to the presence of alternate oxidoreductases (Tokuyama et al., 2014).

Other important by-products in 3-HP production are lactate and acetate. The deletion of *K. pneumoniae ldhA* encoding lactate dehydrogenase was reported to eliminate lactate formation (Kumar et al., 2013b). Another study reported a reduction in lactate formation upon inactivation of *ldh1* and *ldh2* in *K. pneumoniae* (Li et al., 2016). Likewise, to reduce the acetate formation, the synthesis gene *pta* was inactivated, leading to a reduction but not elimination of acetate formation in *K. pneuomoniae* (Li et al., 2016). To further optimize 3-HP producing cell factories, it is essential to assure that by-product formation is reduced to a minimum. Besides the obvious loss of carbon that could have otherwise been used for biomass and product formation, it also leads to a more complex fermentation broth thus potentially increasing costs associated with downstream purification.

The NAD^+^/NADH balance is of importance for the 3-HP production due to the NAD^+^-dependence of the employed aldehyde dehydrogenases. The synthesis of by-products 1,3-PDO and lactate also generates NAD^+^. Elimination of respective pathways thus further skews the NAD^+^/NADH balance (Figure 3). Under aerobic conditions, NAD^+^ is also regenerated by the electron transport chain. However, due to the oxygen-sensitivity of glycerol dehydratase, 3-HP production is often performed under microaerophilic or anaerobic conditions where NAD^+^ regeneration is reduced. To counter these effects, various enzymes can be used to modulate the NAD^+^/NADH balance. In the context of 3-HP production, over-expression of the genes encoding either NADH oxidase, NADH dehydrogenase, or glycerol-3-phosphate dehydrogenase *in K. pneumoniae* showed potential in stimulating 3-HP production via regenerating the NAD^+^ pool in all cases (Li et al., 2013b).

From an economical point of view, it would be beneficial to use a production strain capable of synthesizing coenzyme B_12_. However, even in *K. pneumoniae* which is capable thereof, it has been reported that addition of coenzyme B_12_ increases the glycerol dehydratase activity (Ashok et al., 2013). This could indicate that upregulation of coenzyme B_12_ synthesis is another parameter for increasing the glycerol dehydratase activity. Studies on *Bacillus megaterium* and more recently on *P. denitrificans* show good potential for increasing coenzyme B_12_ synthesis, e.g., by removing the riboswitch-based feedback inhibition system (Biedendieck et al., 2010; Nguyen-Vo et al., 2018).

The application of genome scale metabolic models is a common approach in the field of metabolic engineering and has been used successfully in multiple studies. Using a genome-scale metabolic model for *K. pneumoniae*, no single mutants that would improve 3-HP production were predicted. Instead, a double knockout of *tpi* (triose phosphate isomerase) and *zwf* (glucose-6-phosphate-1-dehydrogenase) involved in central metabolism was suggested. The introduction of these two mutations led to a 4.4-fold increase in the 3-HP yield compared to the parent strain (Tokuyama et al., 2014). Predictions were made of beneficial mutations in *B. subtilis*, but here only the more obvious candidate, glycerol kinase, was suggested as a beneficial mutation (Kalantari et al., 2017).

Another important aspect of optimizing the production strain is to improve the robustness of the strain, specifically, alleviating stress. Probably the most significant stressor is that imposed by the intermediate product 3-HPA and, to a lesser extent, the product 3-HP. Warnecke et al. developed a method to screen for regions and genes that could be involved in the tolerance to 3-HP. Starting with a genomic library of *E. coli* clones, each carrying a plasmid with a genomic DNA insert, they selected for increased tolerance to 3-HP and subsequently quantified the enriched plasmid DNA by microarray analysis (Warnecke et al., 2010). Using this method, they concluded that 3-HP inhibition is due to the limitations in the chorismate and threonine pathways. Surprisingly, they found that over-expression of almost any of the genes in the pathways dramatically alleviated the stress imposed by 3-HP (Warnecke et al., 2010). In a follow up study, the authors identified a 21-amino acid peptide that, when expressed, increased the 3-HP tolerance about 2.3-fold (Warnecke et al., 2012).

The potential of using omics data to identify the targets for improving 3-HP tolerance has been demonstrated. Using 2D gel-based proteomics, Liu and co-workers identified 46 up- and 23 down-regulated proteins upon challenging *E. coli* with 5 g/L of 3-HP. Over-expression of several of these proteins alleviated the stress imposed by 3-HP (Liu et al., 2016).

In recent years, there have been key improvements in the technology for generating targeted libraries of mutants, which is exemplified in a study by Liu and co-workers where a method termed iCREATE for iterative genome editing was used to generate 162,000 mutations in 115 genes (Liu et al., 2018). Using this approach, the production of 3-HP via the Malonyl-CoA pathway was increased 60-fold (Liu et al., 2018). However, for its successful employment, appropriate screening and/or selection techniques need to be in place. Small molecule biosensors based on transcription factors or riboswitches can couple metabolite concentration to a measurable output such as fluorescence for selection or selectable output such as antibiotic resistance (Liu et al., 2017). It is, thus, of particular importance that a 3-HP-inducible system was identified in *P. putida* recently and demonstrated to be functional when expressed in *E. coli* (Hanko et al., 2017). This biosensor has already found application for screening in the directed evolution of the aldehyde dehydrogenase KGSADH which improved the catalytic efficiency of the enzyme 2.8-fold (Seok et al., 2018).

Combining approaches such as iCREATE for generating diversity, and biosensors for detection or selection of improved variants should hold high promises for moving bacterial 3-HP production strains to industrially relevant levels of production.

## Process Engineering for Optimizing 3-HP Production

In a majority of the studies reviewed for this paper, the ability of the recombinant strains to produce 3-HP was tested in 1.5–5 L bioreactors operated in fed-batch mode (Table 3; Supplementary Table 1). A fed-batch operation typically entails growing the cells until the end of the exponential phase. This is followed by the addition of feed medium containing the substrates, thus keeping the growth rate of the microorganism at a desired level (Liu, 2017). A fed-batch mode of operation has certain advantages over batch fermentation. For instance, a fed-batch operation has the ability to prevent overflow metabolism, that is, the phenomenon where fast-growing cells, in the presence of oxygen, utilize fermentation instead of respiration to produce energy (Basan et al., 2015). Another advantage of a fed-batch operation is its ability to maintain the substrate concentration at a low level (de Fouchécour et al., 2018). This is particularly important when the substrate is glycerol. It was reported that no growth of *K. pneumoniae* cells could be seen above 110 and 133 g/L glycerol (extrapolated values) in aerobic and anaerobic conditions, respectively, with glycerol concentrations higher than 40 g/L showing significant growth inhibition (Cheng et al., 2005). Consequently, high titers of 3-HP cannot be obtained from glycerol in a batch fermentation. In *L. reuteri*, the conversion of glycerol to 3-HPA was reported to be about 10 times faster than the subsequent conversion of 3-HPA to 3-HP and 1,3-PDO, resulting in the accumulation of the toxic intermediate 3-HPA. To circumvent this problem, a fed-batch fermentation process was established. It produced 3-HP and 1,3-PDO without any accumulation of 3-HPA. This was achieved by identifying a maximum value of specific glycerol consumption rate at which only 1,3-PDO and 3-HP are produced, and no accumulation of 3-HPA takes place. By maintaining the glycerol amount in the bioreactor such that its specific consumption rate stayed under this maximum value, a 3-HP titer of 14 g/L with a productivity of 0.25 g/L.h was obtained (Dishisha et al., 2014, 2015). This underlines how the knowledge of cell metabolism can be used to solve production problems by means of process engineering.

**Table 3:** Summary of studies reporting the highest 3-HP titer, yield, and productivity in various production hosts.

**Strain**	**Carbon source**	**Fermentation conditions**	**Titer^a^**	**Yield^a^**	**Produc-tivity^a^**	**Reference**
*K. pneumoniae* Δ*ldh1*Δ*ldh2*Δ*pta*_pTAC-*puuC*	Glycerol	5-L bioreactor, fed-batch fermentation, pH 7.0, 37°C, 1.5 vvm air, 400 rpm	83.8	n.a.	1.16	Li et al., 2016
*K. pneumoniae* Kp4 Δ*ldhA*Δ*dhaT*_ *lac*-*aldH*	Glycerol	5-L bioreactor, fed-batch fermentation, pH 7.0, 37°C, 2.2 L/min air, 450 rpm	61.9	0.58	1.62^b^	Jiang et al., 2018
*E. coli* W3110 Δ*ackA-pta*Δ*yqhD*_ *T7*-*dhaB-gdrAB-gabD4*	Glycerol	5-L bioreactor, fed-batch fermentation, pH 7.0, 35°C, 1 vvm air, 500 rpm, vitamin B_12_ added externally	71.9	n.a.	1.8	Chu et al., 2015
*E. coli* W Δ*ackA-pta*Δ*yqhD*_*tac*-*dhaB*-*gdrAB*-*KGSADH*	Glycerol and glucose	5-L bioreactor, fed-batch fermentation, pH 7.0, 37°C, 1 vvm air, 500 rpm, coenzyme B_12_ added externally	40.5	0.97 g/g	1.35	Lim et al., 2016
*L. reuteri* RPRB3007^c^	Glycerol	3-L bioreactor, anaerobic fed-batch fermentation, pH 7.0, 37°C, 500 rpm	10.6	n.a.	1.08	Dishisha et al., 2014
*B. subtilis* 168 trp^+^ Δ*glpK*_*pHyperspank*-*dhaB*-*gdrAB*-*puuC*	Glucose	Shake flask, 37°C, 200 rpm, coenzyme B_12_ added externally	10	0.79 g/g	n.a.	Kalantari et al., 2017
*L. reuteri* DSM 20016 and *G. oxydans* DSM 50049^d^	Glycerol	3-L bioreactor, Step 1: anaerobic fed-batch fermentation, pH 5.5, 37°C, 200 rpm. Step 2: aerobic batch fermentation, pH 5.5, 28°C, 1 L/min air, 800 rpm.	23.6	0.98	n.a.	Dishisha et al., 2015
*K. pneumoniae* and *G. oxydans*^e^	Glycerol	7-L bioreactor, fed-batch fermentation, Step 1: pH 7.0, 37°C, 0.2 vvm air, 150 rpm. Step 2: pH 5.5, 28°C, 0.5 vvm air, 600 rpm.	60.5	0.51	1.12	Zhao et al., 2015

a Unless otherwise mentioned, the units of titer, yield, and productivity are g/L, mol_3−HP_/mol_Glycerol_ and g/L.h, respectively

b Calculated based on the published data

c Mutated catabolite repression element (CRE) in the upstream region of the pdu operon

d L. reuteri converted glycerol to 3-HP and 1,3-propanediol followed by G. oxydans converting 1,3-propanediol to 3-HP

e K. pneumoniae converted glycerol to 1,3-propanediol followed by G. oxydans converting 1,3-propanediol to 3-HP

*adhE, alcohol dehydrogenase; aldA, aldehyde dehydrogenase A (E. coli); aldH, γ-glutamyl-γ-aminobutyraldehyde dehydrogenase (E. coli); aldHk, NAD^+^-dependent homolog of E. coli aldH; CRE, catabolite repression element; dhaB, glycerol dehydratase; dhaR, dhaB reactivating factor in L. brevis; dhaS, putative aldehyde dehydrogenase from B. subtilis; dhaT, 1,3-propanediol oxidoreductase; DO, dissolved oxygen; frdA, succinate dehydrogenase; gabD4, aldehyde dehydrogenase (Cupriavidus necator); gdrA, gdrB, glycerol dehydratase reactivase; glpF, glycerol uptake facilitator protein; glpK, glycerol kinase; KGSADH, α-ketoglutaric semialdehyde dehydrogenase (A. brasilense); ldhA, lactate dehydrogenase; n.a. , not available; pduP, propionaldehyde dehydrogenase; puuC, γ-glutamyl-γ-aminobutyraldehyde dehydrogenase (K. pneumoniae); yqhD, NADPH-dependent aldehyde reductase/alcohol dehydrogenase*.

The challenge concerning the requirement of anaerobic conditions for coenzyme B_12_ generation and aerobic conditions for efficient NAD^+^ regeneration for production of 3-HP from glycerol was tackled by electro-fermentation in a recently published study (Kim et al., 2017). Certain microorganisms possess the ability to deliver electrons to solid electrodes when grown under anaerobic conditions. These exoelectrogens, as these microorganisms are called, can be used to control the redox state in the cells independent of the electron transport chain or fermentation. Electro-fermentation utilizes this property of exoelectrogens to drive the unbalanced fermentations. Anodic electro-fermentation has been used for bioconversion of glycerol to 3-HP using recombinant *K. pneumoniae* over-expressing KGSADH from *A. brasilense*. An electrical potential of + 0.5 V vs. Ag/AgCl was applied to the anode, with 2-hydroxy-1,4-naphthoquinone used for shuttling the electrons between the bacteria and the anode. The transfer of electrons from the bacteria to the anode during anaerobic fermentation led to a decrease in NADH/NAD^+^ ratio in the cells, and hence to an enhanced 3-HP production as compared to the fermentative control (Kim et al., 2017). Although the 3-HP titer reached was low, the study nevertheless demonstrated that it is possible to improve NAD^+^ regeneration under anaerobic conditions. With further optimization, this novel strategy might pave the way for providing new opportunities to enhance 3-HP production from glycerol. It might similarly enhance other bioconversions where there is a need to control the intracellular redox state of a cell factory (Kim et al., 2017; de Fouchécour et al., 2018).

In a fermentation operation, the process parameters such as aeration rate, medium composition, etc. play an important role in determining the final outcome. While there are ample reports on strain development, generally less focus has been put on the optimization of the production conditions. Conversion of glycerol to 3-HP being an oxidative reaction, the aeration rate does affect the final yield of the product (de Fouchécour et al., 2018). In one study, aerobic, micro-aerobic and anaerobic conditions were tested in batch fermentations performed to convert glycerol to 3-HP using recombinant *K. pneumoniae*. They reported that amongst the three conditions tested, the micro-aerobic condition yielded the highest 3-HP titer (2.2 g/L; Zhu et al., 2009). In a more recent study, the transcript levels of the genes encoding the enzymes of the CoA-independent pathway of glycerol conversion to 3-HP and the genes coding for the enzymes of the formate hydrogen lyase pathway were analyzed to understand the intracellular response of *K. pneumoniae* under aerobic, micro-aerobic and anaerobic growth conditions (Huang et al., 2016). The transcription of the glycerol dehydratase operon was downregulated in the presence of oxygen, while aldehyde dehydrogenase-, hydrogenase- and formate dehydrogenase-coding genes were found to be upregulated. The authors suggested that in the presence of oxygen, the formate hydrogen lyase pathway consumed the excess NADH generated due to the over-expression of *aldH* (Huang et al., 2016). In another study aimed at enhancing the coproduction of 3-HP and 1,3-PDO in *K. pneumoniae*, the production of by-products such as lactate, ethanol, succinate, and acetate was reduced by disrupting their synthesis (Ko et al., 2017). However, the disruption of *pta-ackA* gene of the acetate pathway led to a reduction in cell growth, glycerol uptake, and 3-HP and 1,3-PDO production. The authors bypassed the disruption of this gene for reducing acetate production by testing various agitation speeds between 200 and 600 rpm. They reported an increase in 3-HP yield from 0.18 to 0.38 mol_3−HP_/mol_glycerol_, respectively, which could be attributed to an enhanced cell growth rate because of better oxygen transfer at 600 rpm. Although acetate production was not completely abolished, they did manage to reduce it from 163 mM (at 400 rpm) to only 80 mM (at 600 rpm; Ko et al., 2017). Recently, a production of 61.9 g/L 3-HP with a yield of 0.58 mol_3−HP_/mol_glycerol_ in 38 h in a 5 L bioreactor operated in fed-batch mode by using an engineered *K. pneumoniae* strain was reported (Jiang et al., 2018). The aeration rate was reduced to half of the initial rate once the cell biomass OD reached close to the maximum value. Furthermore, using the same strategy, they were able to scale-up the process in a 300 L bioreactor and reported a titer of 54.5 g/L for 3-HP with a yield of 0.58 mol_3−HP_/mol_glycerol_ in 51 h.

The growth medium used for cultivating a microorganism plays an important role in influencing the production process. The current record holder for the highest 3-HP titer, addressed this issue by testing different media for the production of 3-HP in *K. pneumoniae* in shake flasks (Li et al., 2016). A gradient concentration of each component of the media (except CaCl_2_) was individually analyzed. The ameliorated medium enabled an increase of 80.5% in the production of 3-HP. They also tested the effect of pH on 3-HP production and found pH 7.0 to be the most appropriate for 3-HP production (Li et al., 2016). To optimize the growth medium for 3-HP production using *L. reuteri*, 30 different media with variable amounts of sugar beet and wheat processing coproducts (used as the carbon source), yeast extract, tween 80 and vitamin B_12_ were tested. The authors reported an increase in 3-HP production yield by 70 %, accompanied with a decreased 3-HPA titer (Couvreur et al., 2017).

Another way to solve the earlier-mentioned challenge of conflicting requirements for coenzyme B_12_ generation and NAD^+^ regeneration, is to divide the conversion of glycerol to 3-HP into 2 steps. First, glycerol is converted into either 3-HPA or a mixture of 3-HP and 1,3-PDO. The second step involves the conversion of 3-HPA and 1,3-PDO to 3-HP. This two-step strategy was employed in a study where wild-type *L. reuteri* resting cells first converted glycerol to 1,3-PDO and 3-HP in equimolar amounts. In the subsequent step, the supernatant was fed to the resting cells of *Gluconobacter oxydans* which oxidized 1,3-PDO to 3-HP, yielding a 3-HP titer of 23.6 g/L (Dishisha et al., 2015). In a similar study, Zhao et al. employed *K. pneumoniae* in the first step to convert glycerol into 1,3-PDO. In the second step, the resting cells of *G. oxydans* were introduced in the same bioreactor after heat inactivating *K. pneumoniae*. This led to the conversion of 1,3-PDO to 3-HP with a yield of 0.52 mol/mol on glycerol and 0.94 mol/mol on 1,3-PDO, and a 3-HP titer of 60.5 g/L (Zhao et al., 2015). The advantage of using two bioconversion steps is that each uncoupled step can be optimized separately. As attractive as it may appear because of high yields, the industrial implementation of this two-step strategy may not be as cost-effective and economical as a single step fermentation because of higher associated costs. Nevertheless, this strategy could well be a first step for achieving an integrated continuous process for the production of 3-HP, which in addition would be highly desirable because of its low operating costs (de Fouchécour et al., 2018).

## Conclusions and Perspectives

**Figure 4:**
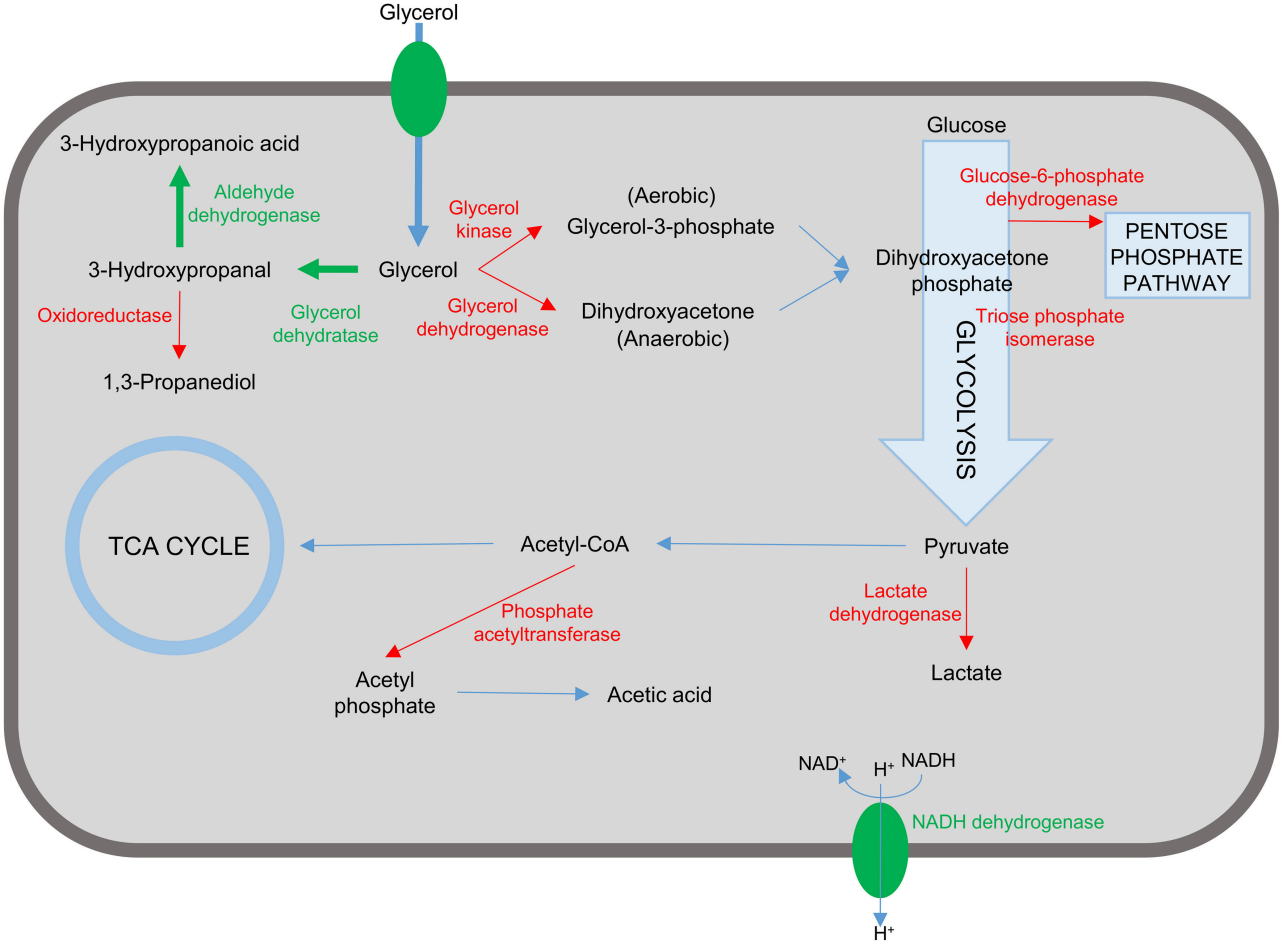
Representation of targets in the bacterial cell for engineering a 3-hydroxypropanoic acid producing strain. Reactions and proteins in green represents targets that are introduced or over-expressed to confer/improve 3-HP production. Unwanted reactions and the corresponding enzymes are shown in red. Inactivation of these targets have been shown to improve 3-HP production and/or reduce/eliminate unwanted by-product formation.

As outlined in this review considering the case of 3-HP production in bacterial cell factories, there are many elements to the successful design of a suitable production strain, ranging from the choice of organism, pathway, and enzymes to engineering of the cell for improved flux through the production pathway and the ability to tolerate the stress imposed by intermediate, by-, and final products (Figure 4). Most of the studies described in this review have targeted some of these elements. The highest titer recorded so far is 83.8 g/L which was achieved in *K. pneumoniae* by optimizing the expression of aldehyde dehydrogenase in the production pathway and increasing flux through the production pathway by blocking synthesis of the by-products lactic acid and acetic acid (Li et al., 2016). This impressive feat was based on rational design and accomplished by combining some of the successful approaches outlined above in very few engineering steps.

For bacterial 3-HP production to reach a level of economic viability, further improvements in titer, and productivity will be necessary. By now, many different enzyme variants have been used for constructing 3-HP production pathways but in many cases the enzymes have not been characterized biochemically. A better understanding of the properties of the enzymes with respect to catalytic properties, pH range, and stability could prove instrumental in identifying the optimal enzymes and would also help to identify enzyme qualities to improve by enzyme engineering.

The impressive body of research on strain engineering has identified numerous challenges and solutions such as balancing enzyme activities, preventing by-product formation and alleviating stresses. With this accumulating knowledge base pertaining to 3-HP cell factory design, it seems likely that it will be possible to further tweak production by combining all of the successful approaches described herein. To facilitate this work, powerful engineering techniques are continually emerging that allow the design of large combinatorial libraries of targeted mutations. Notably, the recent development of a 3-HP biosensor can be used in combination with adaptive laboratory evolution for identifying as-of-yet unknown mechanisms for further fine-tuning of the production strains. While it seems reasonable that further improvements in titer will be achieved in the coming years, there should be an increased focus to also develop industrially relevant process designs and subsequent upscaling in order to provide proof of concept.

In conclusion, the massive developments in synthetic biology and metabolic engineering could be expected to soon deliver the long-sought goal of converting the abundant by-product glycerol into the much desired value-added chemical 3-HP.

## Author Contributions

CJ, AG, and AK performed literature search and drafted the manuscript. All authors revised the manuscript and approved the final version.

### Conflict of Interest Statement

The authors declare that the research was conducted in the absence of any commercial or financial relationships that could be construed as a potential conflict of interest.
