# Supplementary material for: Production of 3-Hydroxypropanoic Acid From Glycerol by Metabolically Engineered Bacteria
Source: Front Bioeng Biotechnol. 2019 May 24;7:124. doi: 10.3389/fbioe.2019.00124 (PMC6542942; doi:10.3389/fbioe.2019.00124)
Supplement: Supplementary file 1 [file Data_Sheet_1.PDF]

Supplementary Table 1. Summary of studies reporting 3-HP production using glycerol as substrate in various production hosts. *adhE*: alcohol dehydrogenase; *aldA*: aldehyde dehydrogenase A (*E. coli*); *aldH*: γ-glutamyl-γ-aminobutyraldehyde dehydrogenase (*E. coli*); *aldHk*: NAD^+-dependent homolog of *E. coli aldH*; *CRE*: catabolite repression element; *dhaB*: glycerol dehydratase; *dhaR*: dhaB reactivating factor in *L. brevis*; *dhaS*: putative aldehyde dehydrogenase from *B. subtilis*; *dhaT*: 1,3-propanediol oxidoreductase; *DO*: dissolved oxygen; *frdA*: succinate dehydrogenase; *gabD4*: aldehyde dehydrogenase (*C. necator*); *gdrA*, *gdrB*: glycerol dehydratase reactivase; *glpF*: glycerol uptake facilitator protein; *glpK*: glycerol kinase; *KGSADH*: α-ketoglutaric semialdehyde dehydrogenase (*A. brasilense*); *ldhA*: lactate dehydrogenase; *n.a.*: not available; *pduP*: propionaldehyde dehydrogenase; *puuC*: γ-glutamyl-γ-aminobutyraldehyde dehydrogenase (*K. pneumoniae*); *yqhD*: NADPH-dependent aldehyde reductase/alcohol dehydrogenase.

**Strain**	**Carbon source**	**Fermentation conditions**	**Titer**[a]	**Yield**[a]	**Productivity**[a]	**References**
K. pneumoniae AK strain expressing PduP (from *L. reuteri*)	Glycerol	5-L bioreactor, batch fermentation, pH 7.0, 37 °C, 0.5 vol/min air	1.38	n.a.	0.05	Luo et al. 2011
K. pneumoniae ME-308_*tac-aldA*	Glycerol	3-L bioreactor, batch fermentation, pH 7.0, 37 °C, micro-aerobic, 300 rpm agitation	2.8	0.1	0.12	Zhu et al. 2009, Kumar et al. 2013A
K. pneumoniae KpCΔ*dhaT_lac-puuC*	Glycerol	5-L bioreactor, fed-batch fermentation, pH 7.0, 37 °C, 1.0 vvm air, 150 rpm agitation, DO < 0.2 %	16	0.23	0.67	Ashok et al. 2011, Kumar et al. 2013A
K. pneumoniae AK_*lacZ-aldHk*	Glycerol	5-L bioreactor, fed-batch fermentation, pH 7.0, 37 °C, 0.5 vol/min air, 200 rpm agitation	6.8	n.a.	0.14	Luo et al. 2011B[f]
K. pneumoniae/pUC18*kan-aldHec_ lacZ-aldH*	Glycerol	5-L bioreactor, anaerobic fed-batch fermentation, pH 7.0, 37 °C, 0.4 vvm nitrogen, 400 rpm agitation	24.4	0.18	1.02	Huang et al. 2012
K. pneumoniae/pUC18*kan-aldHec_ lacZ-aldH*	Glycerol	5-L bioreactor, fed-batch fermentation, pH 7.0, 37 °C, 1.5 vvm air, 400 rpm agitation	48.9	0.41	1.75	Huang et al. 2013[g]
K. pneumoniae KpKΔ*dhaT_ lac-KGSADH*	Glycerol	1.5-L bioreactor, fed-batch fermentation, pH 7.0, 37 °C, 0.3 vvm air, 150 rpm agitation	16.3	0.4	0.3	Ko et al. 2012
K. pneumoniae KpBCΔ*dhaT*Δ*yqhD*_lac-puuC_*tac-dhaB123-gdrAB*	Glycerol	5-L bioreactor, fed-batch fermentation, pH 7.0, 37 °C, constant 5 % DO	28.1	0.4	0.58	Ashok et al. 2013
K. pneumoniae KpCΔ*glpK*Δ*dhaT_ lac-puuC*	Glycerol	5-L bioreactor, anaerobic fed-batch fermentation, pH 7.0, 37 °C, 150 rpm agitation	22.0	0.3	0.46	Ashok et al. 2013B[h]
K. pneumoniae KpKWT_ *lac-puuC*	Glycerol	1.5-L bioreactor, anaerobic fed-batch fermentation, pH 7.0, 37 °C, 250 rpm agitation	11.3	0.27	0.94	Kumar et al. 2012[i]

K. pneumoniae KpK*ΔldhA_ lac-KGSADH*	Glycerol	1.5-L bioreactor, fed-batch fermentation, pH 7.0, 37 °C, 0.2 vvm air, 250 rpm agitation	22.7	0.35	0.38	Kumar et al. 2013B
K. pneumoniae Kp(pET-pk-*dhaS*)*_pk-dhaS*	Glycerol	5-L bioreactor, fed-batch fermentation, pH 7.0, 37 °C, 1.5 vvm air, 400 rpm agitation	18.5	n.a.	0.77	Su et al. 2015
K. pneumoniae J2B *ΔldhAΔfrdAΔadhE_P10-dhaB123-gdrAB_ lac-KGSADH*	Glycerol	1.5-L bioreactor, fed-batch fermentation, pH 7.0, 37 °C, 1 vvm air, 400 rpm agitation	43.0	n.a.	0.9	Ko et al. 2017
K. pneumoniae *Δldh1Δldh2Δpta_*pTAC-*puuC*	Glycerol	5-L bioreactor, fed-batch fermentation, pH 7.0, 37 °C, 1.5 vvm air, 400 rpm agitation	83.8	n.a.	1.16	Li et al. 2016
K. pneumoniae Kp4 *ΔldhAΔdhaT_ lac-aldH*	Glycerol	5-L bioreactor, fed-batch fermentation, pH 7.0, 37 °C, 2.2 L/min air, 450 rpm agitation	61.9	0.58	1.62[b]	Jiang et al. 2018
K. pneumoniae Kp4 *ΔldhAΔdhaT_ lac-aldH*	Glycerol	300-L bioreactor, fed-batch fermentation, two-stage aeriation, pH 7.0, 37 °C	54.5	0.43	1.06[b]	Jiang et al. 2018
K. pneumoniae DSM 2026*_tac-puuC*	Glycerol	5-L bioreactor, fed-batch fermentation, pH 7.0, 37 °C, 1.5 vvm air, 400 rpm agitation	36.7	41.7%	1.02	Wang et al. 2018[j]
E. coli SH254_paraBAD*_araBAD-dhaB_T7-aldH*	Glycerol	Shake flask, aerobic (200 rpm)	0.6	0.48	0.02	Raj et al. 2008
E. coli SH-BGA1*_ T7-dhaB-gdrAB_ T5-aldH*	Glycerol	5-L bioreactor, fed-batch fermentation, pH 7.0, 37 °C, 0.35 vvm air, 500 rpm agitation	31.0	0.35	0.43	Mohan Raj et al. 2009[k], Kumar et al. 2013A
E. coli SH-BGK1*_ T7-dhaB-gdrAB-KGSADH*	Glycerol	5-L bioreactor, fed-batch fermentation, pH 7.0, 37 °C, 0.35 vvm air, 500 rpm agitation	38.7	0.35	0.54	Rathnasingh et al. 2009, Kumar et al. 2013A
E. coli JHS00947*_ T7-dhaB-dhaR-aldH*	Glycerol and glucose	2.5-L bioreactor, fed-batch fermentation, pH 6.8, 25 °C, 1 vvm air, 1200 rpm agitation	14.3	0.15	0.26	Kwak et al. 2013
E. coli BL21(DE3)*_ T7-dhaB-gdrAB_tac-KGSADH*	Glycerol	Shake flask, 37 °C, 200 rpm	5.1	n.a.	n.a.	Niu et al. 2017[l]
E. coli W3110 (DE3) *ΔackA-ptaΔyqhDΔglpR_ T7-dhaB-gdrAB-aldH-glpF*	Glycerol	5-L bioreactor, fed-batch fermentation, pH 7.0, 35 °C, 1 vvm air, 500 rpm agitation	42.1	0.268 g/g	1.32	Jung et al. 2014
E. coli W3110 *ΔackA-ptaΔyqhD_ T7-dhaB-*	Glycerol	5-L bioreactor, fed-batch fermentation, pH 7.0, 35 °C, 1 vvm air, 500 rpm agitation	71.9	n.a.	1.8	Chu et al. 2015

gdrAB-gabD4							
E. coli W Δ*ackA-pta*Δ*yqhD_tac-dhaB-gdrAB-KGSADH*	Glycerol and glucose	5-L bioreactor, fed-batch fermentation, pH 7.0, 37 °C, 1 vvm air, 500 rpm agitation	40.5	0.97 g/g	1.35	Lim et al. 2016
E. coli W*_SP4-dhaB_SP3-gdrAB_SP5-KGSADH*	Glycerol and glucose	1.5-L bioreactor, fed-batch fermentation, pH 7.0, 37 °C, 1 vvm air, 650 rpm agitation	56.4	n.a.	1.18	Sankaranarayanan et al. 2017[m]
E. coli BL21*_T7-dhaB-gdrAB-KGSADH*	Glycerol and glucose	5-L bioreactor, fed-batch fermentation, pH 6.5, 37 °C, 1 vvm air, 450 rpm agitation	17.2	n.a.	0.34[b]	Niu et al. 2017B[n]
E. coli W_ *tac-dhaB-gdrAB_lacP-KGSADH*	Glycerol	1.5-L bioreactor, fed-batch fermentation, pH 7.0, 37 °C, 0.5 vvm air, 650 rpm agitation	41.5	31%	0.86	Sankaranarayanan et al. 2014
E. coli BW24113_ *PLlacO1- dhaB-gdrAB-KGSADH*	Glycerol	Shake flask, 37 °C, 150 rpm	6.06	n.a.	0.13	Tsuruno et al. 2015[o]
L. reuteri RPRB3007[c]	Glycerol	3-L bioreactor, anaerobic fed-batch fermentation, pH 7.0, 37 °C, 500 rpm agitation	10.6	n.a.	1.08	Dishisha et al. 2014
L. reuteri RPRB3007[c]	Glycerol	Anaerobic fed-batch fermentation with immobilized cells, 37 °C, 100 rpm agitation	3.3	0.48	0.09	Zaushitsyna et al. 2017[p]
B. subtilis 168 trp$^+$ Δ*glpK_pHyperspank-dhaB-gdrAB-puuC*	Glucose	Shake flask, 37 °C, 200 rpm	10	0.79 g/g	n.a.	Kalantari et al. 2017
L. reuteri DSM 20016 and *G. oxydans* DSM 50049[d]	Glycerol	3-L bioreactor, Step 1: anaerobic fed-batch fermentation, pH 5.5, 37 °C, 200 rpm agitation. Step 2: aerobic batch fermentation, pH 5.5, 28 °C, 1 L/min air, 800 rpm agitation	23.6	0.98	n.a.	Dishisha et al. 2015
K. pneumoniae and *Gluconobacter oxydans*[e]	Glycerol	7-L bioreactor, fed-batch fermentation, Step 1: pH 7.0, 37 °C, 0.2 vvm air, 150 rpm agitation. Step 2: pH 5.5, 28 °C, 0.5 vvm air, 600 rpm agitation	60.5	0.51	1.12	Zhao et al. 2015

[a]Unless otherwise mentioned, the units of titer, yield and productivity are g/L, mol$_{3\text{-}HP}$/mol$_{Glycerol}$ and g/L.h respectively

[b]Calculated based on the published data

[c]Mutated catabolite repression element (CRE) in the upstream region of the *pdu* operon

[d]*L. reuteri* converted glycerol to 3-HP and 1,3-propanediol followed by *G. oxydans* converting 1,3-propanediol to 3-HP

[e]*K. pneumoniae* converted glycerol to 1,3-propanediol followed by *G. oxydans* converting 1,3-propanediol to 3-HP

[f]Luo L, et al (2011). J Ind Microbiol Biotechnol 38: 991-999.

[g]Huang Y et al (2013). Bioresour Technol 128: 505-512.

[h]Ashok S, et al. (2013B). Metabolic Engineering 15: 10-24.

[i]Kumar V et al (2012). Appl Microbiol Biotechnol 96: 373-383.
[j]Wang J, et al (2018). Microb Cell Fact 17: 56.
[k]Mohan Raj S, et al (2009). Appl Microbiol Biotechnol 84: 649-657
[l]Niu K, et al (2017A). 3 Biotech 7(5): 314.
[m]Sankaranarayanan M, et al (2017). J Biotechnol 259: 140-147.
[n]Niu K, et al (2017B). Biotechnol Appl Biochem 64: 572-578.
[o]Tsuruno K, et al (2015). Microb Cell Fact 14: 155.
[p]Zaushitsyna O, et al (2017). Journal of Biotechnology 241: 22-32.
